# Reviewing the current state of legacy POP-brominated flame retardants in plastic childcare products and toys: a scoping review protocol

**DOI:** 10.1186/s13643-024-02524-1

**Published:** 2024-06-03

**Authors:** Rebecca Mlelwa, Hanna-Andrea Rother

**Affiliations:** Environmental Health Division and Centre for Environmental and Occupational Health Research, School of Public Health, University of Cape Town, Observatory, Cape Town, 7925 South Africa

**Keywords:** Brominated flame retardants, Toys, Childcare products, Recycling, Plastics, Circular economy, Stockholm Convention, Policy

## Abstract

**Background:**

Due to their adverse environmental and health impacts, brominated flame retardants (BFRs) are listed in Annex A of the Stockholm Convention for global elimination of production and use. Their health impacts include endocrine disruption, cancer, reproductive effects, and neurobehavioral and developmental disorders in children. Emerging literature suggests that legacy POP-BFRs are increasingly found in consumer products, including those used for and by children. The presence of legacy POP-BFRs in children’s products is a big concern. Children are more vulnerable to chemical exposure risks than adults because their bodies are still developing and fragile. The rising problem is contributed to by the global push towards a circular economy that encourages responsible production and consumption by practising the recycling of waste materials. Waste materials such as electronic and electrical equipment plastics often contain POP-BFRs. POP-BFRs in waste materials are transferred into new products through recycling. The recycled products have become a potential source of exposure to legacy POP-BFRs for vulnerable populations, particularly children. Our scoping review aims to map and summarise the emerging literature. This information is needed to inform evidence-based policies to protect children from toxic exposures.

**Methods:**

Our scoping review will follow a methodological framework proposed by Arksey and O’Malley. Peer-reviewed and grey literature on the topic will be retrieved from electronic databases and other relevant sites. Two reviewers will screen titles and abstracts, followed by a full-text review of studies for eligibility based on the established inclusion and exclusion criteria. Data will be extracted, and findings will be mapped in a table according to study settings, types of children’s products tested, and concentration of legacy POP-BFRs in contaminated products. A map chart will be created to display how contaminated products are spread globally.

**Discussion:**

Because of their unique vulnerabilities, children continue to suffer disproportionate exposures to toxic chemicals compared to adults. Information on potential exposures, particularly for children, is crucial to make evidence-based policies. We intend to map and summarise the emerging literature on legacy POP-BFRs in children's products. Findings will be disseminated to relevant stakeholders through publishing in a peer-reviewed scientific journal and policy briefs.

**Systematic review registration:**

The protocol is registered with the Open Science Framework (10.17605/OSF.IO/7KDE5).

**Supplementary Information:**

The online version contains supplementary material available at 10.1186/s13643-024-02524-1.

## Background

The Stockholm Convention bans persistent organic pollutants (POPs) to protect the environment and human health [1]. Brominated flame retardants (BFRs), including polybrominated diphenyl ethers (PBDEs), tetra-bromo bisphenol A (TBBP-A), and hexabromocyclododecane (HBCD) are among the toxic chemicals listed in Annex A of the Stockholm Convention for global elimination of production and use since 2009 [1]. These chemicals are classified as persistent organic pollutants (POPs). There are well-documented environmental and health impacts linked to BFRs [2]. The impacts include endocrine disruption [3], reproductive effects [4], cancer [5], and neurobehavioral and developmental disorders in children [6].

PBDEs, TBBP-A, and HBCD, also called POP-BFRs, have been used extensively in electronic and electrical equipment (EEE) casings, polyurethane foam, textiles, vehicles, and construction materials since the 1970s [7]. Plastics constitute a significant portion of the EEE and vehicles [8]; hence, a substantial proportion of EEE waste is generated globally. Due to their strength, versatility, and thermal properties, WEEE plastics are often recycled into new, cheap consumer products. The current global push towards a circular economy contributes to recycling WEEE plastics into new products [9].

Although circular economy promotes resource efficiency [10] and contributes towards attaining sustainable development goals (SDGs), particularly SDG 12 on responsible consumption and production, it also threatens SDG 3 on good health and well-being for all in some ways. The WEEE plastics usually contain legacy POP-BFRs [11]. Due to limited technologies, screening and separating legacy POP-BFRs from the WEEE plastics before recycling is still a challenge facing the recycling sector [12]. As a result, toxic chemicals in WEEE plastics are transferred into the new products through recycling [13]. Recycled products have become a potential source of consumers' exposure to legacy toxic chemicals such as POP-BFRs. The circular economy approach thus poses a unique emerging public health challenge.

About 50% of WEEE from the European Union (EU), Canada, and Australia is shipped illegally to China, India, Nigeria and Ghana [11]. Reports in China show that about 30% of consumer products, such as toys and food-contact utensils, are made from recycled materials [14]. China is a top toy manufacturer globally, supplying about 70% of the global market [15]. Many countries are flooded with cheap toys from China [16]. This suggests that contaminated products are widely spread globally.

Furthermore, several countries, including Brazil, Canada, Japan, Cambodia, the Republic of Korea and Turkey [17], have been granted exemption under the Stockholm Convention to recycle materials containing POP-BFRs despite the evidence of environmental and health impacts caused by these chemicals. The recycling exemption was introduced in 2009, soon after banning POP-BFRs and is listed in Part IV and V of Annex A of the Stockholm Convention. The agreement to establish these exemptions was reached during the parties' fourth conference (COP-4) and is applicable until 2030 [18]. Because of the exemptions, the circulation of legacy POP-BFRs in material value chains will likely continue for many years.

The emerging literature indicates that legacy POP-BFRs are detected in recycled products at alarmingly high levels. This is happening despite current legislative measures such as Low POP content levels (LPCLs) of 1000 mg/kg for a sum of PBDEs and 100 mg/kg for HBCD 50 mg/kg in waste materials. The LPCLs are established in the Basel and the Stockholm Conventions, and they are cut-off limits at which materials in the waste stream should be considered hazardous POP-waste and must be dealt with with strict measures [19].

A survey conducted in Europe found that 73% of samples of food-contact products and toys made of recycled plastic contained PBDEs and TBBP-A at concentrations ranging from 200 to 10,000 mg/kg [20]. Another study in China reported legacy POP-PBDEs at a concentration ranging from 0.45 to 21.30 mg/kg in plastic products such as washbasins, mops, and children’s play mats [21]. Similar reports from low-and-middle-income countries (LMICs) [9, 22–25] also exist. Other reported consumer products include kitchen utensils [22] and childcare products such as changing table pads, toilet seats, car seats, hair accessories, and toys [9, 20, 23, 24].

The presence of legacy POP-BFRs in products meant for children is a huge concern. Children have unique vulnerabilities because they undergo a growing phase. Their body organs are still developing and fragile [25]. Their bodies cannot detoxify toxic chemicals during this period due to immature metabolic systems [26]. Also, their behavioral patterns, such as putting toys into their mouth, increase their exposure to the legacy POP-BFRs in these products. Even at low doses, exposure to toxic chemicals during childhood can impact children's health in the short and long term [27]. Children have a longer lifespan compared to adults and are likely to get more exposure over time [28].

Understanding the extent to which legacy POP-BFRs are detected in children’s products is paramount. Such information is essential for establishing evidence-based policies to protect vulnerable groups, particularly children. However, no studies have been undertaken to map the emerging literature and summarize the evidence.

## Aim

Our scoping review aims to map the emerging literature regarding legacy POP-BFRs contamination in childcare products and toys. We expect the scoping review will reveal the magnitude of the problem and uncover the literature gaps for further research. Findings will help influence policy decisions to stop recycling legacy POP-BFRs into new products, especially products intended for children's use.

## Methodology

The scoping review will be conducted to map the available literature and summarise findings [29]. The scoping review is appropriate for this topic as it gives flexibility to explore general questions and related literature rather than answering a focused question [30]. It will help to know the extent of available literature on this topic, synthesize it, and provide a general overview [31].

Our scoping review will follow the methodological framework proposed by Arkesey and O’Malley [32] and advanced by Levac et al. [33]. The methodology employs the following steps: (i) identifying the research question, (ii) identifying relevant studies, (iii) selecting eligible studies, (iv) charting the data, and (v) collating, summarising, and reporting the results.

The Preferred Reporting Items for Systematic Reviews and Meta-analyses (PRISMA) Extension for Scoping Reviews (PRISMA-ScR) [34] will be followed when writing the scoping review. This scoping review protocol was developed following the PRISMA-ScR checklist (see Additional file 1: PRISMA-ScR checklist). The protocol is registered with the Open Science Framework (10.17605/OSF.IO/7KDE5).

### Stage 1: Identify the research question

Based on the preliminary literature searches, we hypothesize that products made of recycled plastics are contaminated with POP-BFRs through recycling practices. Our scoping review seeks to answer the main question, ‘What is the evidence that plastic childcare products and toys are contaminated with legacy POP-BFRs?.’ The specific questions for this scoping review are the following:

1. To what extent are legacy POP-BFRs detected globally in plastic childcare products and toys?
2. Which types of plastic childcare products and toys have been tested, and what are the documented levels of POP-BFRs in the tested samples compared to the LPCLs?
3. Which types of POP-BFRs are detected in plastic childcare products and toys?

### Stage 2: Identifying relevant studies

Identification of relevant studies will be done by applying a search strategy. To develop a search strategy, we first conducted a preliminary search on PubMed to identify relevant studies. We then used keywords from the relevant studies to develop a full search strategy with the help of an experienced librarian at Bongani Mayosi Health Sciences Library at the University of Cape Town.

The full search strategy included specific Medical Subject Headings (MeSH) terms and keywords following the Population-Concept-Context (PCC) framework recommended by the Joanna Briggs Institute (JBI) [35]. These include “halogenated diphenyl ethers,” “brominated flame retardants,” “childcare products,” “toys,” and “plastic.” The keyword “brominated flame retardants” was expanded to include specific names “polybrominated diphenyl ethers,” “decabromodiphenyl ethers,” “pentabromodiphenyl ethers,” “octabromo diphenyl ether,” “tetra-bromobisphenol-A,” and “hexabromocyclododecane,” to ensure no crucial information is left out. The librarian peer-reviewed the full search strategy using the Peer Review of Electronic Search Strategies (PRESS) checklist, as recommended by McGowan and colleagues in their PRESS guidelines for systematic reviews and other evidence syntheses [36].

We piloted the search strategy on PubMed to check whether it retrieves a reasonable number of records as targeted [37]. The search strategy was later refined accordingly for searching in other appropriate databases, including Web of Science, Ebscohost, Scopus, and Cochrane. Full search strings for the different databases and the pilot search results are included as Additional file 2. A reference list of identified articles will be reviewed for additional sources.

Grey literature, such as unpublished reports by relevant organizations and government documents, will be searched in Google Scholar, OpenGrey, WorldWideScience, and OpenDoar using the keywords “halogenated diphenyl ethers,” “brominated flame retardants,” “childcare products,” “toys,” and “plastic.” Websites of relevant organizations will also be reviewed for reports. Examples of relevant organizations include the International Pollutants Elimination Network (IPEN), a network of over 600 organizations working on eliminating hazardous chemicals.

### Stage 3: Study selection

#### Eligibility

The eligibility of the published and grey literature on this topic will depend on the following inclusion and exclusion criteria:

#### Inclusion criteria

- Primary studies, both published and unpublished, explore plastic toys and childcare products. Such products include eating utensils, washbasins, play mats, toilet seats, hair accessories, etc. Studies that report products used by children as well as products used by adults will be eligible. However, due to the scope of this scoping review, only the chemical concentration findings of children’s products will be captured during data extraction.
- Primary studies, both published and unpublished, that explore the POP-BFRs listed in the Stockholm Convention (i.e., polybrominated diphenyl ethers (PBDEs), tetra-Bromo bisphenol A (TBBP-A) and hexabromocyclododecane (HBCD).
- Primary studies with quantitative methods will be included. A reference list of review articles will be screened to check whether there are relevant primary studies that meet the inclusion criteria.
- Published and unpublished primary studies in English.
- No limit will be set on the publication date.

#### Exclusion criteria

- Primary studies, both published and unpublished, that explore non-plastic materials.
- Primary studies, both published and unpublished, that report products not used for and by children.
- Published and unpublished primary studies that report chemicals other than the POP-BFRs.
- Published and unpublished primary studies in languages other than English.
- Reviews, opinions, and commentaries will be excluded.

**Fig. 1:**
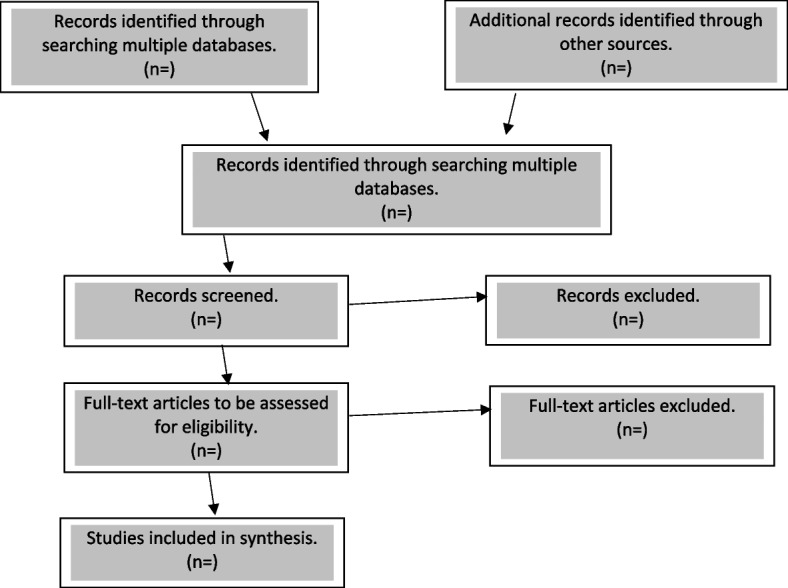
Study selection flow diagram recommended in the Preferred Reporting Items for Systematic Reviews and Meta-Analyses Extension for Scoping Reviews (PRISMA-ScR)

A decision tree will be created and used during the screening process to ensure a consistent and efficient screening process following the established inclusion and exclusion criteria [37]. The identified literature will be imported into Endnote to locate and remove duplicates. After that, a two-staged screening will be done by two reviewers using reference management software Rayyan [38]. The first stage will involve screening titles and abstracts based on the pre-established criteria to ascertain whether the articles meet the criteria. Stage two will involve a full-text review. The reference list of the eligible studies will be explored to identify additional articles. Discussions will be used to resolve disagreements if they arise between the two reviewers, and a third reviewer will be involved for unresolvable conflicts between the two reviewers. The searching and screening results will be reported in full in the final scoping review according to the Preferred Reporting Items for Systematic Reviews and Meta-Analyses Extension for Scoping Reviews (PRISMA-ScR) [34] and illustrated in a PRISMA-ScR flow diagram (see Fig. 1).

### Stage 4: Charting the data

A data extraction form (see Additional file 3) will be used to extract data from the identified literature in line with the aim of the proposed scoping review. The data extracted will include bibliographic information such as authors’ names, year of publication, country, and study title. We will also capture the types of tested children’s products, the sample size, the country of origin, and the types and levels of POP-BFRs detected in the tested products. The data extraction form was piloted by two reviewers and checked by the third reviewer. It will be revised and modified as necessary during the data extraction process. The final scoping review will detail any modifications to the data extraction form. The two independent reviewers will perform data extraction and any disagreements will be resolved by involving the third reviewer.

### Stage 5: Collating, summarising, and reporting results

Extracted data will be presented in a tabular form to respond to the review questions as recommended by JBI guidelines for scoping review protocols [35]. Research question 1 (*RQ1: To what extent are POP-BFRs detected in plastic childcare products and toys globally?)* will be approached by summarising the percentages of samples contaminated with POP-BFRs as reported in eligible studies. We will create a map chart in Excel using frequency counts of eligible studies to display their geographical location and show the extent to which POP-BFRs-contaminated toys and childcare products are spread globally. The country of origin of the contaminated products will also be presented in the table in case such information is reported in the eligible studies. For research question 2 *(RQ2: Which types of plastic childcare products and toys have been tested, and what are the documented levels of POP-BFRs in the tested samples compared to the LPCLs?),* types of reported products such as, e.g., toys, eating utensils, washbasins, play mats, toilet seats, and hair accessories will be listed in a column in the table together with the sample sizes. Levels of POP-BFRs detected in the products will be presented in the table per the reported concentrations (mg/kg or ppm). The POP-BFRs categories (*RQ3: Which types of POP-BFRs are detected in plastic childcare products and toys?)* will be classified as octaBDE, decaBDE, HBCD, TBBPA, and ΣBFRs where relevant. A narrative summary will accompany the tabulated data and will explain how the findings relate to the review questions. We will also explain the significance of the collected evidence, highlight any gaps, and draw conclusions based on the aims of this scoping review.

## Discussion

Because of their unique vulnerabilities, children continue to suffer disproportionate exposures to environmental hazards such as toxic chemicals compared to adults. The ever-rising number of cases of chronic diseases in children proves this. More so are the adult life chronic diseases originating from childhood exposures. Yet efforts to reduce children’s exposure to toxic chemicals are hindered by practices such as recycling legacy POP-BFRs into new products. Information about potential exposures is essential for making informed decisions, yet such information is often insufficient or lacking. Our scoping review will help to fill the information gaps by summarising the emerging evidence and identifying needs for further research. This will offer an opportunity to develop and propose recommendations for protecting children from further exposure to legacy contaminants. Findings will be disseminated through publishing in a peer-reviewed scientific journal. Additionally, a policy brief will be disseminated to relevant stakeholders. Popular social media channels will be used to communicate findings to the general public.

### Supplementary Information

Additional file 1: PRISMA-ScR checklist

Additional file 2: Search strategy

Additional file 3: Data extraction form

## Data Availability

Data sharing is not applicable to this article as no datasets were generated or analyzed during the current study.

## Abbreviations

BFRs: Brominated flame retardants
COP: Conference of parties
HBCD: Hexabromocyclododecane
IPEN: International pollutants elimination network
LPCLs: Low POP concentration limits
LMICs: Low-and-middle income countries
MeSH: Medical subject headings
PRISMA-P: Preferred reporting items for systematic reviews and meta-analyses protocols
PRISMA-ScR: Preferred reporting items for systematic reviews and meta-analyses (PRISMA) extension for scoping reviews
POPs: Persistent organic pollutants
PBDEs: Polybrominated diphenyl ethers
PCC: Population-concept-context
PRESS: Peer review of electronic search strategies
TBBP-A: Tetra-bromo bisphenol A
