# Supplementary material for: Reviewing the current state of legacy POP-brominated flame retardants in plastic childcare products and toys: a scoping review protocol
Source: Syst Rev. 2024 Jun 3;13:148. doi: 10.1186/s13643-024-02524-1 (PMC11149179; doi:10.1186/s13643-024-02524-1)
Supplement: Supplementary file 2 — Additional file 2: Search strategy [file 13643_2024_2524_MOESM2_ESM.docx]

**Additional file 2**

| **Search Strategy** | **# of hits pilot search retrieved March 2024** |
| --- | --- |
| **Full search strategy for PubMed**  ((("Halogenated Diphenyl Ethers"[Mesh]) OR ("polybrominated diphenyl ethers" OR "polybrominated diphenyl ether" OR PBDE OR PBDEs OR "Decabromodiphenyl ethers" OR "Decabromodiphenyl ether" OR DecaBDE OR decabdes OR "Pentabromodiphenyl ether" OR "Pentabromodiphenyl ethers" OR PentaBDE OR PentaBDEs OR "octabromo diphenyl ether" OR "octabromo diphenyl ether" OR OctaBDEs OR OctaBDE OR "Tetra- bromobisphenol-A" OR TBBP-A OR hexabromocyclododecane OR HBCD OR Bromine)) AND (("Play and Playthings"[Mesh]) OR (toy OR toys OR "childcare product" OR "childcare products" OR "childcare utensil" OR "childcare utensils" OR "children article" OR "childcare articles" OR "consumer product" OR "consumer products" OR plaything OR playthings OR "child product" OR "children products" OR "childhood product" OR "childhood products" OR "infant product" OR "infants products" OR "baby product" OR "babies products" OR "toddler products" OR "girl toys" OR "girls toys" OR "boy toy" OR "boys toys" OR "kid toy" OR "kids toys" OR "youngster toy" OR "youngsters toys" OR "preschooler toys" OR "school-age children toys" OR "school-aged children toys" OR "schoolchildren toys"))) AND ("Plastics"[Mesh])  **Limits:** English language | **41** |
| **Full search strategy for Web of Science**  (TS= ("halogenated diphenyl ether*" OR "polybrominated diphenyl ether*" OR pbde* OR "Decabromodiphenyl ether*" OR decabde* OR "Pentabromodiphenyl ether*" OR pentabde* OR "Octabromodiphenyl ether*" OR octabde* OR "tetra-bromobisphenol-a" OR tbbp-a OR "hexabromocyclododecane" OR hbcd)) AND (TS=(toy* OR "childcare product*" OR "childcare utensil*" OR "children article*" OR "consumer product*" OR plaything* OR "child product*" OR "childhood product*" OR "infant* product*" OR "baby product*" OR "babies product*" OR "toddler product*" OR "girl* toy*" OR "boy* toy*" OR "kid* toy*" OR "youngster* toy*" OR "preschooler toy*" OR "school-age children toy*" OR "schoolchildren toy*")) AND (TS=plastic*)  **Limits:** English language | **134** |
| **Full search strategy for Scopu**s  (TITLE-ABS-KEY("halogenated diphenyl ether*" OR "polybrominated diphenyl ether*" OR pbde* OR "Decabromodiphenyl ether*" OR decabde* OR "Pentabromodiphenyl ether*" OR pentabde* OR "Octabromodiphenyl ether*" OR octabde* OR "tetra-bromobisphenol-a" OR tbbp-a OR "hexabromocyclododecane" OR hbcd)) AND (TITLE-ABS-KEY(toy* OR "childcare product*" OR "childcare utensil*" OR "children article*" OR "consumer product*" OR plaything* OR "child product*" OR "childhood product*" OR "infant* product*" OR "baby product*" OR "babies product*" OR "toddler product*" OR "girl* toy*" OR "boy* toy*" OR "kid* toy*" OR "youngster* toy*" OR "preschooler toy*" OR "school-age children toy*" OR "schoolchildren toy*")) AND (TITLE-ABS-KEY(plastic*))**Limits:** English language | **82** |
| **Full search strategy for EBSCOhost**  *TX("halogenated diphenyl ether*" OR "polybrominated diphenyl ether*" OR pbde* OR "Decabromodiphenyl ether*" OR decabde* OR "Pentabromodiphenyl ether*" OR pentabde* OR "Octabromodiphenyl ether*" OR octabde* OR "tetra-bromobisphenol-a" OR tbbp-a OR "hexabromocyclododecane" OR hbcd) AND TX(toy* OR "childcare product*" OR "childcare utensil*" OR "children article*" OR "consumer product*" OR plaything* OR "child product*" OR "childhood product*" OR "infant* product*" OR "baby product*" OR "babies product*" OR "toddler product*" OR "girl* toy*" OR "boy* toy*" OR "kid* toy*" OR "youngster* toy*" OR "preschooler toy*" OR "school-age children toy*" OR "schoolchildren toy*") AND TX(plastic*)*  **Limits:** English language | **149** |
| **Full search strategy for Cochrane**  ( "halogenated diphenyl ether*" OR "polybrominated diphenyl ether*" OR pbde* OR "Decabromodiphenyl ether*" OR decabde* OR "Pentabromodiphenyl ether*" OR pentabde* OR "Octabromodiphenyl ether*" OR octabde* OR tetra-bromobisphenol-a OR tbbp-a OR hexabromocyclododecane OR hbcd ) in All Text AND ( toy* OR "childcare product*" OR "childcare utensil*" OR "children article*" OR "consumer product*" OR plaything* OR "child product*" OR "childhood product*" OR "infant* product*" OR "baby product*" OR "babies product*" OR "toddler product*" OR "girl* toy*" OR "boy* toy*" OR "kid* toy*" OR "youngster* toy*" OR "preschooler toy*" OR "school-age children toy*" OR "schoolchildren toy*" ) in All Text AND ( plastic*) - (Word variations have been searched) | **4** |
| **Full search strategy for Google Scholar**  ( halogenated diphenyl ether OR "polybrominated diphenyl ether*" OR "Decabromodiphenyl ether*" OR "Pentabromodiphenyl ether*" OR "Octabromodiphenyl ether*" OR tetra-bromobisphenol-a OR hexabromocyclododecane)AND ( toy* OR "childcare product*" OR "childcare utensil*" OR "children article*" OR "consumer product*" OR plaything* OR "child product*" OR "baby product*" OR "babies product*" OR "girl* toy*" OR "boy* toy*" OR "kid* toy*" OR "youngster* toy*" OR "preschooler toy*" OR "school-age children toy*" OR "schoolchildren toy*" ) AND (plastic*) AND (report* OR “government document”) | **82** |
