# Supplementary material for: Reviewing the current state of legacy POP-brominated flame retardants in plastic childcare products and toys: a scoping review protocol
Source: Syst Rev. 2024 Jun 3;13:148. doi: 10.1186/s13643-024-02524-1 (PMC11149179; doi:10.1186/s13643-024-02524-1)
Supplement: Supplementary file 3 — Additional file 3: Data extraction form [file 13643_2024_2524_MOESM3_ESM.docx]

**Data extraction template**

Reviewer:……………………………..

| **Study Country/ies** | **Types of products tested for POP-BFRs** | **Number of samples (N)** | **Percentage (n/N) of POP-BFRs-positive samples, detected POP-BFRs and concentration (range) in ppm, mg/kg or ng/g** | | | | | | | **Comments (source of data (e.g., figure/table/page number, any assumptions/transformation/calculations made etc)** | **Reference** |
| --- | --- | --- | --- | --- | --- | --- | --- | --- | --- | --- | --- |
|  |  |  | **OctaBDE mixture (BDE 153, 154, 183, 196, 197, 203, 206, and 207)** | **PentaBDE mixture (BDE 28, 47, 49, 66, 85, 99, and 100)** | **DecaBDE mixture (BDE 209)** | **ΣPBDEs** | **HBCDD** | **TBBPA** | **ΣBFRs** |  |  |
|  |  |  |  |  |  |  |  |  |  |  |  |
|  |  |  |  |  |  |  |  |  |  |  |  |
|  |  |  |  |  |  |  |  |  |  |  |  |
|  |  |  |  |  |  |  |  |  |  |  |  |
|  |  |  |  |  |  |  |  |  |  |  |  |
|  |  |  |  |  |  |  |  |  |  |  |  |
|  |  |  |  |  |  |  |  |  |  |  |  |
|  |  |  |  |  |  |  |  |  |  |  |  |
|  |  |  |  |  |  |  |  |  |  |  |  |
|  |  |  |  |  |  |  |  |  |  |  |  |
|  |  |  |  |  |  |  |  |  |  |  |  |
|  |  |  |  |  |  |  |  |  |  |  |  |
|  |  |  |  |  |  |  |  |  |  |  |  |
|  |  |  |  |  |  |  |  |  |  |  |  |
|  |  |  |  |  |  |  |  |  |  |  |  |
|  |  |  |  |  |  |  |  |  |  |  |  |
|  |  |  |  |  |  |  |  |  |  |  |  |
